# Recurrent Idiopathic Cerebral Venous Thrombosis

**DOI:** 10.7759/cureus.86167

**Published:** 2025-06-16

**Authors:** Melissa Tebaldi, Zackary Anderson, Darion Lago, Keegan Plowman, Carl Ruthman

**Affiliations:** 1 Internal Medicine, Naples Comprehensive Health, Naples, USA; 2 Pulmonary and Critical Care, Naples Comprehensive Health, Naples, USA

**Keywords:** beta thalassemia minor, cerebral venous thrombosis, ischemic injury, mechanical thrombectomy (mt), venous thrombosis

## Abstract

A 40-year-old female with a past medical history of beta thalassemia minor presented to the ED with an intractable headache, nausea, and vomiting. A CT head without IV contrast was performed that was negative for any signs of subarachnoid hemorrhage, or thrombotic etiology. The patient presented 48 hours later and was found to have cerebral venous thrombosis (CVT). She was subsequently taken for mechanical thrombectomy. Despite mechanical thrombectomy, her intracranial pressure (ICP) continued to rise. The patient underwent neurosurgical intervention with placement of external ventricular drain (EVD). Despite EVD placement, pressure continued to rise intracranially. The patient then went for a repeat thrombectomy, which was unsuccessful as clots continued to reaccumulate. No existing tool or measurement can lead to better outcomes regarding CVT cases. It is possible that with improved imaging modalities, early diagnosis is possible with high clinical suspicion, but further data needs to be collected. Per our literature review, there is no existing data regarding reaccumulating of clot burden despite intervention. This case represents the need for a broader discussion about risk stratification to identify quantifiable measures for better patient outcomes.

## Introduction

Cerebral venous thrombosis (CVT) is a disease typically affecting young adults, women, and children. Per American Heart Association (AHA) studies, there is an incidence of two and five per million per year [1]. CVT in the idiopathic form represents 12.5% of all cases of CVT [1]. Typically, these cases present in women of childbearing age with unremitting pain described as the “worst headache of life,” which is concerning for subarachnoid hemorrhage. These patients present with symptoms related to obstruction, including thunderclap headache, visual loss, and seizure. The first step in management is a CT head without IV contrast [2]. In the setting of CVT, this imaging study is often negative. However, indirect signs, such as venous congestion, may lead to an increased suspicion of venous thrombosis. There is an estimated 34.2% mortality rate despite improvements in treatment and diagnostic therapies [2]. With such a high mortality rate, the clinical diagnosis and early detection of CVT are crucial in overall survivorship. Here, we demonstrated the case of a young 40-year-old female with idiopathic CVT with delayed onset detection. This article was previously presented as a meeting abstract at the 2023 American Federation for Medical Research (AFMR) National Meeting on October 27, 2023.

## Case presentation

**Figure 1:**
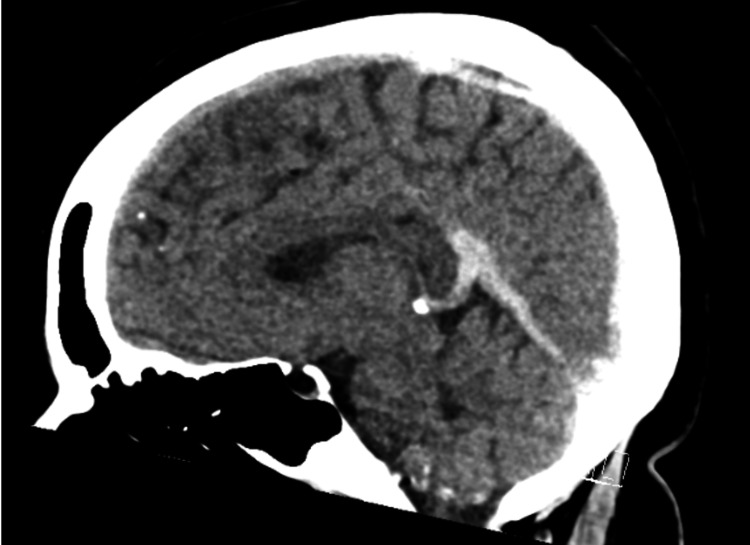
Sagittal View of Head CT Imaging showing hyperdense straight sinus and dorsal aspect of sagittal sinus compatible with thrombus.

**Figure 2:**
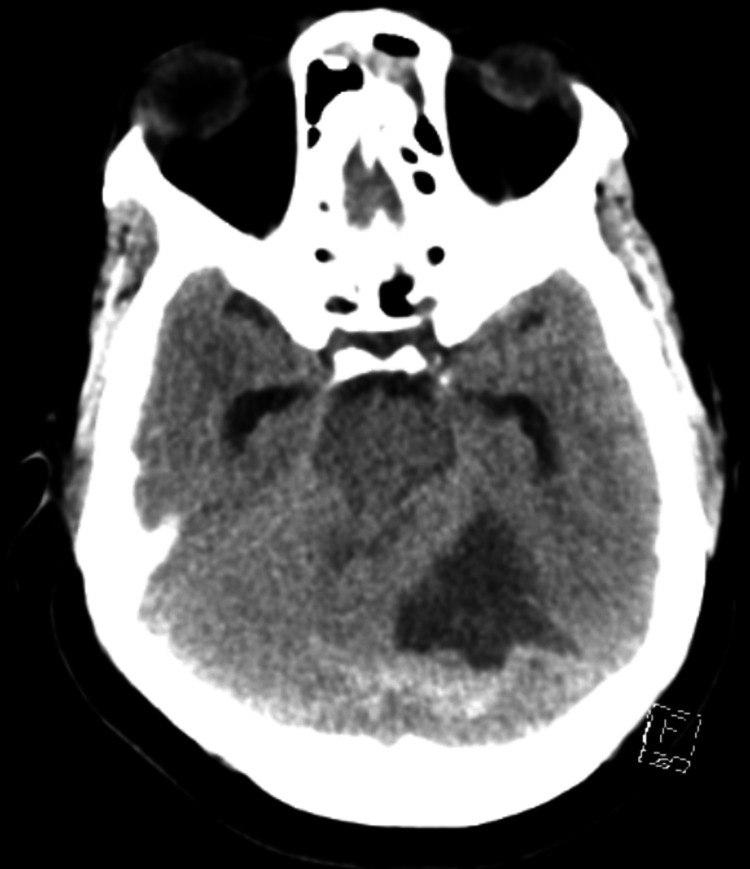
Axial View of Head CT Imaging showing obstructive hydrocephalus with effacement of the fourth ventricle and dilated lateral and third ventricles.

**Figure 3:**
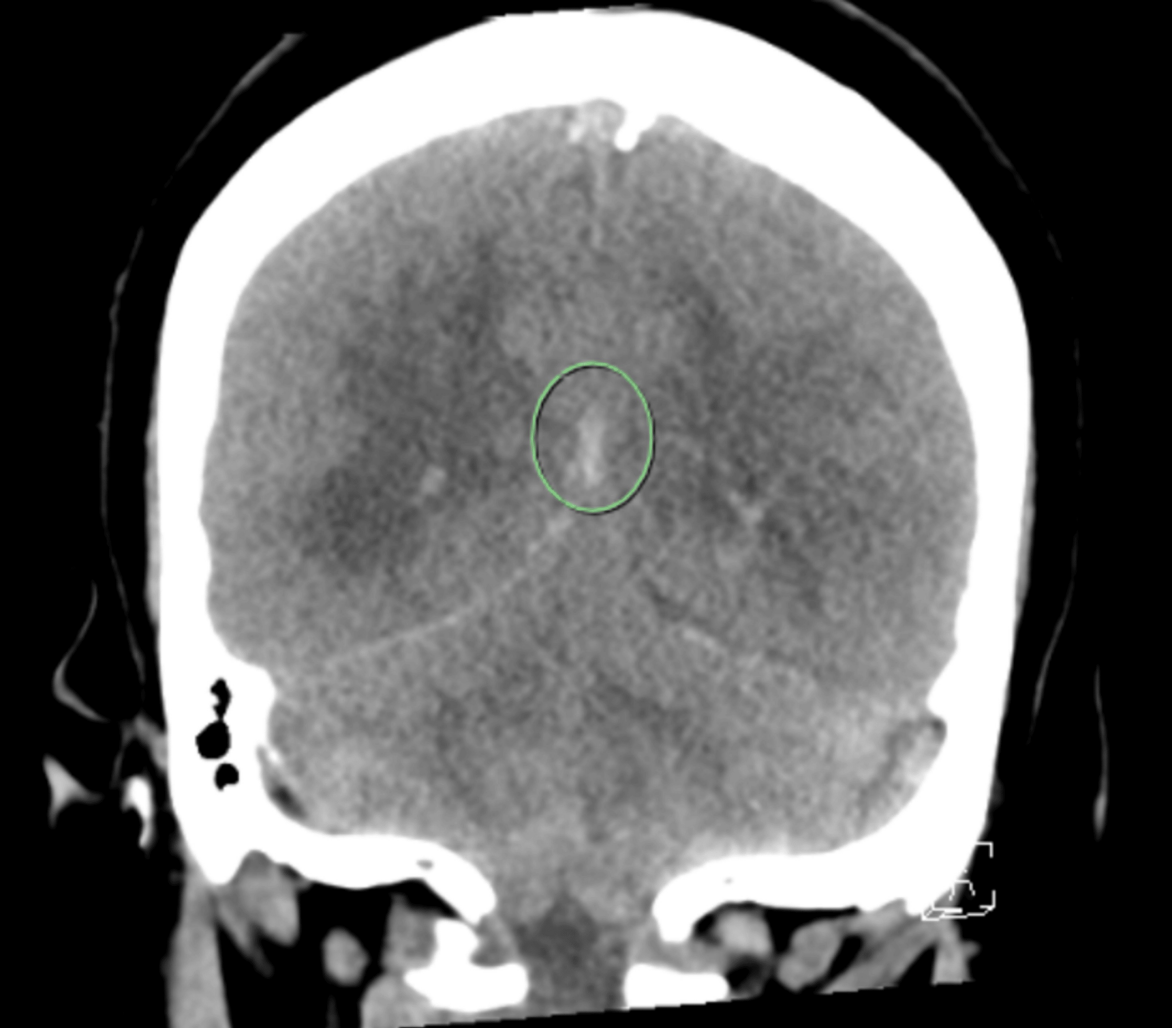
Coronal View of Head CT Imaging demonstrating subarachnoid hemorrhage.

**Figure 4:**
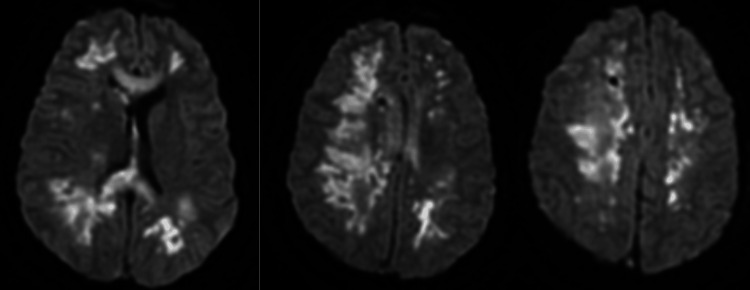
Brain MRI Imaging demonstrating multiple watershed supratentorial and basal ganglia infarcts and left temporal lobe hemorrhage.

A 40-year-old female with a past medical history of thalassemia presented with an intractable headache, nausea, vomiting, and profound lethargy. Prior to the patient’s arrival, she had an unremarkable medical history. She had no outstanding medical history outside of beta thalassemia trait. She had never had any blood clots nor any incidence of strokes, thrombosis, or deep vein thrombosis in her family. She was not on any medications or supplements and did not smoke. In the emergency room, a neurological exam showed poor response to verbal and tactile stimuli. Glasgow Coma Scale (GCS) was three, and she was subsequently intubated. CT venogram demonstrated a thrombus of the bilateral transverse sinus and superior aspect of the left jugular vein (Figure 1). The patient was taken to interventional radiology with the successful removal of a large amount of clot burden, and she was started on a heparin infusion. The following day, her neurological examination worsened with minimal pain response in all extremities, nonreactive right pupillary response, weak cough reflex, and absent corneal reflexes. Repeat CT head showed the development of obstructive hydrocephalus (Figure 2). The heparin infusion was held, and an external ventricular drain (EVD) was placed to manage elevated intracranial pressure (ICP). She was given a single dose of mannitol, started on hypertonic 3% saline, and maintained at serum sodium levels above 150. Despite EVD, mannitol, and hypertonic 3% saline, ICP remained elevated between 40 and 80 mmHg. Due to these high ICPs, the patient was taken back to interventional radiology for invasive angiography and was found to have rapid re-accumulation of venous thrombosis. Thrombectomy was once again performed, removing a significant amount of clot burden. Post-thrombectomy care focused on maintaining adequate cerebral perfusion pressure (CPP) in the setting of her continually elevated ICP. MRI was notable for multiple acute/subacute, supratentorial infarcts. A follow-up CT venogram showed large areas of attenuation in the right middle cerebral artery (MCA) territory with persistent hypodensity in the straight sinus, which is concerning for yet another episode of re-thrombosis (Figure 3). Due to concern for a hypercoagulable state, the patient underwent workup for infection, lupus anticoagulant, prothrombin, factor V Leiden, prothrombin, anticardiolipin, and B2 glycoprotein, and malignancy with CT chest, abdomen, and pelvis, all of which were negative for any acute findings. At this point, the patient was managed medically with heparin as she was deemed at high risk for intracranial hemorrhage if another invasive procedure was performed. Subsequent MRI imaging showed a worsening neuro prognosis with the progression of multiple watershed infarcts with loss of flow-void involving sinuses compatible with venous sinus thrombosis (Figure 4). Despite ongoing medical management, the patient was unable to survive her significant neurological damage.

## Discussion

CVT is a rare disorder with significant mortality and morbidity that is often not considered as a part of an initial differential. When patients typically present with a thunderclap, the worst headache of their life presentation, it often leads to a non-contrast CT of the head as more common etiologies such as ischemic stroke and subarachnoid hemorrhage have this association. As we ruled out hemorrhage and more common etiologies, with these imaging studies, the severity of the symptoms should warrant someone to investigate further when they meet a certain patient demographic. 

Although rare, the pathophysiology behind CVT is believed to be due to decreased cerebral perfusion, which causes an ischemic injury and results in intracellular edema. This edema is believed to disrupt the blood-brain barrier, leading to vasogenic edema and subsequent hemorrhage [3]. Typical presentations of CVT include headache, intracranial hypertension, subarachnoid hemorrhage with focal neurological deficits, and/or unexplained seizures. This usually occurs in women of childbearing age, most often in the setting of a peripartum or postpartum state, hypercoagulable states such as pregnancy, hormone replacement therapy, oral contraceptives, and inherited thrombophilia such as antithrombin deficiency, factor V Leiden, peripheral thrombin genetic mutation 201010, as well as hyperhomocysteinemia and uremia [4]. The presence of these prothrombin gene mutations combined with other risk factors warrants additional tests [5]. Uncommon causes of CVT in recent years have been associated with infections, reported cases including heparin-induced thrombocytopenia (HIT) and thrombotic thrombocytopenic purpura (TTP) [6,4], predisposing patients to an increased risk. Per AHA guidelines, a plain CT or MRI can be useful in the initial evaluation of patients with suspected CVT. However, a negative CT or MRI does not rule out CVT. If suspicion for CVT is high, a CT-venogram or MR-venogram should be performed [2,6]. Patients with evidence of increased ICP should be closely monitored for progressive neurological changes [6]. Antiepileptic therapy should be considered if the patient displays any seizure-like activity. Without evidence of seizure activity, it is not recommended to initiate antiepileptic drugs. Patients should be started on anticoagulation [2,7]. If the patient continues to have neurological deterioration, endovascular intervention or decompressive hemicraniectomy can be performed [6]. If there are concerns for infection, patients should also be started on antibiotics, and a lumbar puncture can be considered.

Despite these measures, mortality rates in these cases remain high. The treatment of CVT is controversial, and more studies need to be performed as conflicting data in the literature exist [8,9]. In one study, the highest predictors of death on physical exam were found in cases of patients who had impaired consciousness, seizures, and mental status disorders [9]. In this same study, the size of parenchymal lesions (detailed as greater than 5 cm) was associated with a worsening mortality rate [9]. Regarding early diagnosis and treatment, data remain controversial [8-10]. In various retrospective studies, prompt and early initiation of anticoagulation and treatment have shown favorable outcomes when a high clinical suspicion and prompt anticoagulation occurs [8,11].

Despite aggressive treatment on the second admission, although subjective, it can be assumed that our patient was not presenting within the initial stages of her initial infarct, as the physical exam was vastly different on the second arrival. Our patient was experiencing seizures and worsening mental status, which have been shown to be the worst predictors of death in CVT [9].

With that in mind, data conflicts on whether early clinical diagnosis and prompt anticoagulation can lead to better patient outcomes. Although it is unclear if early intervention can make a difference, altered mental status (AMS), seizures, and lesion size are associated with worse outcomes. In addition, consistently, studies show that increased mortality when outcomes are associated with AMS, right hemisphere hemorrhage, and deep venous thrombosis [10]. The highest risk of death occurs due to trans-tentorial herniation, thus prompting early intervention [2,7,10]. 

With limited data and cases, further studies are needed for further quantifiable results to achieve better outcomes. No existing tool or measurement can lead to better outcomes regarding CVT cases. It is possible that with improved imaging modalities, early diagnosis is possible with high clinical suspicion, but further data needs to be collected [8]. Per our literature review, there is no existing data regarding reaccumulating clot burden despite intervention. This case represents the need for a broader discussion about risk stratification to identify quantifiable measures for better patient outcomes.

## Conclusions

CVT is associated with significant morbidity and mortality in young patient populations. Symptoms can often be overlooked or associated with more common etiologies such as ischemic strokes or subarachnoid hemorrhages. This can be further complicated by delays in seeking medical attention, as appropriate testing is often not ordered. CVT should be considered in the differential diagnosis of thunderclap headache, seizure, and AMS, in the right patient population. This case represents the need for a broader discussion about risk stratification to identify quantifiable measures for better patient outcomes.
